# Conjugated oligo (phenylene vinylene) covalently linked porphyrin for sonodynamic therapy

**DOI:** 10.1002/smo.20240035

**Published:** 2024-11-13

**Authors:** Wenhua Jia, Junqing Wang, Ling Li, Qiong Yuan, Yuze Wang, Xinyi Zhang, Yanli Tang

**Affiliations:** ^1^ Key Laboratory of Analytical Chemistry for Life Science of Shaanxi Province Key Laboratory of Applied Surface and Colloid Chemistry Ministry of Education School of Chemistry and Chemical Engineering Shaanxi Normal University Xi'an China

**Keywords:** antitumor agents, conjugated oligomer, porphyrin, sensitizers, sonodynamic therapy

## Abstract

Sonodynamic therapy (SDT) is garnering considerable attention as a promising treatment for deep‐seated tumors because of its strong tissue penetration ability, non‐invasiveness, and controllability. However, the SDT efficiency of traditional sonosensitizers including porphyrins and their derivatives are limited due to their poor water dissolubility, high aggregation, and low reactive oxygen species (ROS) production efficiency. Consequently, it is crucial to develop novel sonosensitizers with high yields of ROS, outstanding water solubility, and good biocompatibility. Herein, we constructed a new platform for SDT based on unimolecular porphyrin derivatives OPV‐C_3_‐TPP. The probe OPV‐C_3_‐TPP was synthesized by covalently linking conjugated oligomers (OPV) with 5, 10, 15, 20‐tetra (4‐aminophenyl) porphyrin (TAPP). The introduction of OPV greatly improves the water solubility of the porphyrins and reduces the self‐aggregation of the porphyrins. In addition, OPV‐C_3_‐TPP has good intramolecular energy transfer efficiency, thus enhancing the yield of ROS. The experimental results show that OPV‐C_3_‐TPP exhibits excellent ROS generation capacity under ultrasound (US) irradiation, which leads to apoptosis and necrosis of tumor cells. In vivo tumor growth is also significantly inhibited in the OPV‐C_3_‐TPP + US group, exhibiting better SDT effects than TAPP. Therefore, the unimolecular OPV‐C_3_‐TPP can be used as a potential sonosensitizer, providing a promising SDT for deep‐tissue tumors.

## Graphical abstract

The probe OPV‐C_3_‐TPP by covalently linking conjugated oligomers (OPV) with 5, 10, 15, 20‐tetra (4‐aminophenyl) porphyrin (TAPP) was reported for sonodynamic therapy (SDT). The introduction of OPV greatly enhanced the yield of reactive oxygen species under ultrasound irradiation, which leads to apoptosis and necrosis of tumor cells. OPV‐C3‐TPP + US significantly inhibited in vivo tumor growth, exhibiting better SDT efficiency than TAPP.

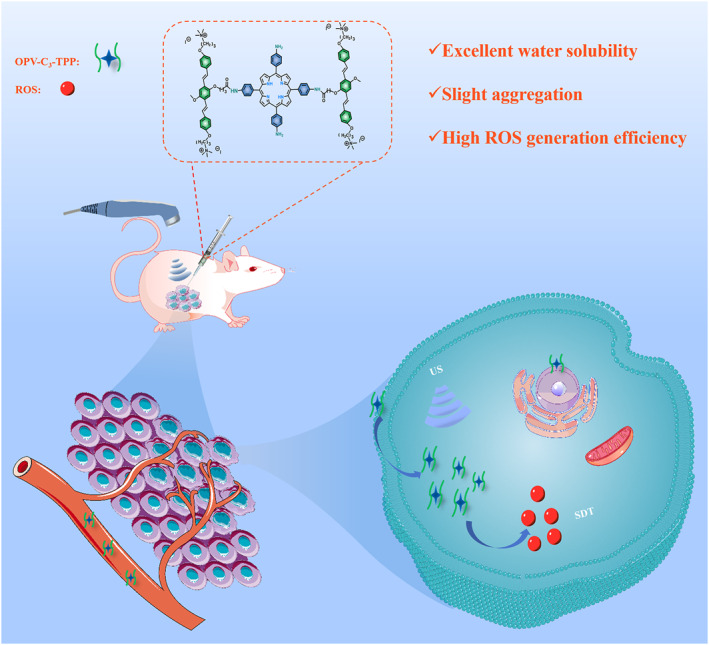

## 1 INTRODUCTION

Cancer is one of the malignant diseases that poses a serious threat to public health.^[1]^ Therefore, it is urgent to develop noninvasive and safe cancer therapeutic method to achieve excellent therapeutic efficiency and negligible side effects. In recent years, more efforts have been taken to the alternative treatment, including photothermal therapy,[^2^, ^3^] photodynamic therapy,[^4^, ^5^, ^6^] bringing more possibilities to cancer treatment. Phototherapy uses light to stimulate the production of reactive oxygen species (ROS) from photosensitizers or heat energy from photothermal agents for killing cancer cells. However, the penetration depth of light is limited, bringing a critical challenge for phototherapy in biomedical applications.^[7]^

In the 1980s, Yumita and his colleagues found that hematoporphyrin could be used to kill tumor cells, which showed high cytotoxicity under ultrasound (US) irradiation, proposing it as sonodynamic therapy (SDT).^[8]^ SDT requires three basic conditions, including US, oxygen and sonosensitizer. It is generally believed that in the presence of oxygen, external US can make the sonosensitizer produce ROS through the cavitation effect, inducing cell apoptosis, necrosis and autophagy, and finally wrecking tumor cells.[^9^, ^10^] SDT is becoming an emerging non‐invasive tumor therapeutic modality due to its distinctive and superior properties, including minimal side effects, high penetration depth, noninvasiveness, high therapeutic efficiency and excellent biocompatibility.[^11^, ^12^, ^13^, ^14^, ^15^]

Sonosensitizers have played a vital role to determine the therapeutic effect in SDT.[^9^, ^16^] The ideal sonosensitizers commonly behave the following three aspects: excellent reactive oxygen generation ability, good water solubility and biocompatibility.^[17]^ Basically, sonosensitizers include two categories, organic sonosensitizer and inorganic sonosensitizer. Inorganic sonosensitizers such as Au NPs, titanium dioxide NPs, graphene, and mesoporous silica nanoparticles have good physical and chemical properties and higher stability,[^18^, ^19^, ^20^, ^21^, ^22^, ^23^, ^24^, ^25^, ^26^] however, the generation efficiency of ROS is relatively low. In addition, the poor biocompatibility and non‐biodegradability of these inorganic nanomaterials severely limit their clinical applications.

In contrast, most organic sonosensitizers originated from photosensitizers offer distinctive advantages: (1) explicit molecular structure, clear synthesis route and good reproducibility; (2) high ROS generation efficiency; and (3) good biocompatibility and degradability.^[27]^ The earliest and frequently employed organic sonosensitizers were porphyrins and their derivatives,[^28^, ^29^, ^30^, ^31^, ^32^, ^33^, ^34^] for example, protoporphyrin IX,[^35^, ^36^] hematoporphyrin (Hp) and hematoporphyrin monomethyl ether,[^37^, ^38^, ^39^] etc. Under US irradiation, porphyrins can produce ROS, indicating potential sonosensitizers in SDT. Several organic small molecules with low toxicity and good therapeutic efficacy have also been identified as sonosensitizers.[^40^, ^41^, ^42^, ^43^, ^44^] Although porphyrins and other molecules showed well‐defined molecular structural features, these compounds exhibited low stability, poor water solubility, high aggregation, and low ROS production efficiency, which hindered their clinical application in the field of SDT. Therefore, it is essential to exploit novel sonosensitizers with excellent water solubility, high SDT efficiency, and good biocompatibility.

**FIGURE 1:**
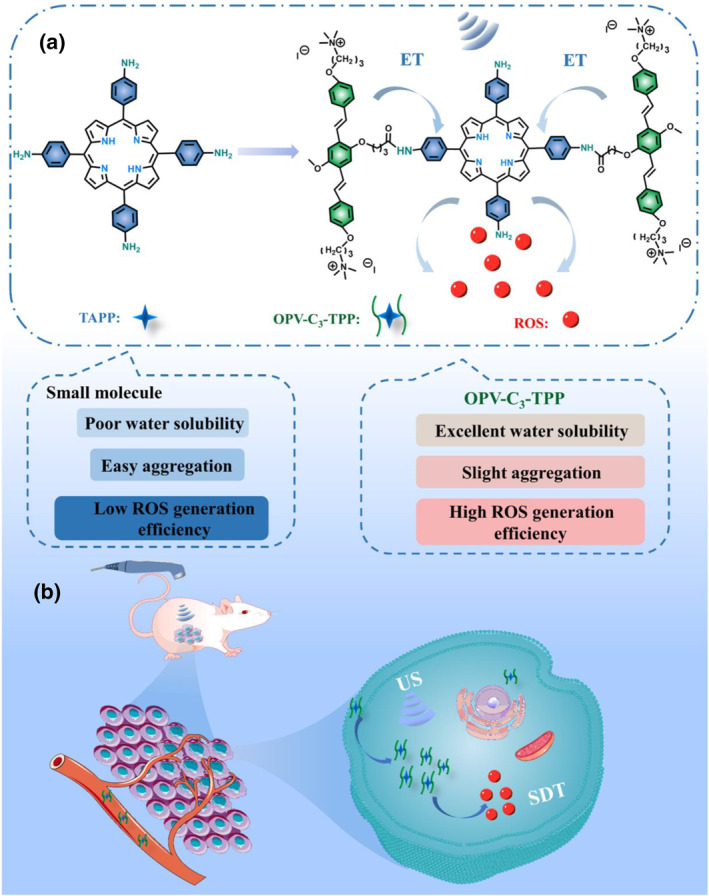
(a) Chemical structure and excellent properties of the unimolecular porphyrin derivatives OPV‐C_3_‐TPP in sonodynamic therapy; (b) schematic illustration of OPV‐C_3_‐TPP in the sonodynamic therapy.

Conjugated oligomers have received considerable attention because of their clear molecular structures, great light‐harvesting ability and tunable optoelectronic properties.[^45^, ^46^, ^47^, ^48^, ^49^] In this work, we developed a new strategy based on a unimolecular probe for SDT to overcome poor water solubility and easy aggregation of porphyrin derivatives. The unimolecular porphyrin derivatives (OPV‐C_3_‐TPP) were obtained by covalently linking water‐soluble cationic conjugated oligo‐(phenylenevinylene) (OPV, donor) and 5, 10, 15, 20‐tetra (4‐aminophenyl) porphyrin (TAPP, acceptor) (Figure 1). The covalent connection between OPV and TAPP not only enhanced the water solubility of porphyrin but also lessened the self‐aggregation effect of porphyrin. Because a good overlap was observed between the emission spectrum of OPV and the absorption spectrum of porphyrin, OPV‐C_3_‐TPP showed excellent energy transfer efficiency and enhanced the yield of active oxygen. Furthermore, OPV‐C_3_‐TPP showed excellent in vitro and in vivo SDT effects under US irradiation.

## 2 RESULTS AND DISCUSSION

OPV‐C_3_‐TPP was first synthesized according to the procedure reported in our previous literature.^[33]^ In addition, the photophysical properties of OPV‐C_3_‐TPP were determined in aqueous solution, which showed a maximum absorption wavelength of 427 nm and a maximum emission wavelength of 460 nm. Furthermore, OPV‐C_3_‐TPP showed three weak absorption peaks in the 500–700 nm band and an emission peak of porphyrin at 650 nm (Figure S1), demonstrating the typical photophysical properties of TAPP. The above results indicated that OPV was successfully covalently linked to the porphyrin molecular structure.

**FIGURE 2:**
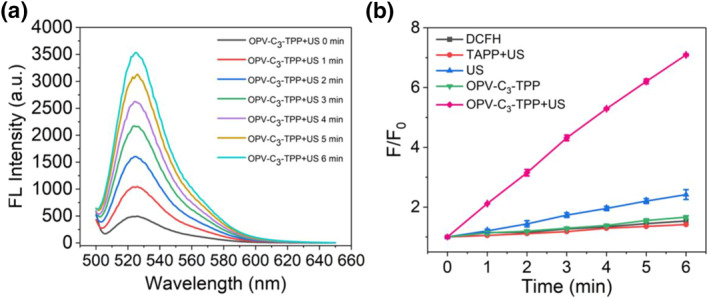
ROS production of OPV‐C_3_‐TPP under US. (a) Fluorescence spectra of DCFH in the presence of OPV‐C_3_‐TPP under US irradiation. (b) Fluorescence intensity ratio of DCFH in the presence of OPV‐C_3_‐TPP or TAPP with or without US irradiation. DCFH, 2′,7′‐dichlorodihydrofluorescein; ROS, reactive oxygen species; TAPP, 5, 10, 15, 20‐tetra (4‐aminophenyl) porphyrin; US, ultrasound.

To estimate the sonodynamic ability of OPV‐C_3_‐TPP, we initially employed 2′,7′‐dichlorodihydrofluorescein diacetate (DCFH‐DA) as an ROS probe in vitro. Under alkaline conditions, DCFH‐DA is converted to the non‐fluorescent compound DCFH. Then, in the presence of ROS, it is oxidized to fluorescent 2′,7′‐dichlorofluorescein (DCF). Thus, ROS generation can be assessed by measuring the fluorescence intensity of DCF at 525 nm under excitation at 488 nm. As shown in Figure 2a, in the presence of OPV‐C_3_‐TPP, the fluorescence of DCF gradually enhanced with the increase in US irradiation time, suggesting that OPV‐C_3_‐TPP could produce ROS triggered by US stimulation. It is noteworthy that OPV‐C_3_‐TPP has a higher ROS generation efficiency in comparison with DCFH solution without OPV‐C_3_‐TPP (control group) under the US irradiation. The final fluorescence intensity of the OPV‐C_3_‐TPP solution was increased approximately 7.1‐fold compared to the initial fluorescence intensity. In addition, under the same conditions, there was no significant change in the fluorescence intensity of the solution in the TAPP + US group, indicating that no ROS were generated from TAPP (Figure 2b). The above results indicated that the covalent modification of cationic conjugated oligomers on the porphyrin greatly improved the SDT of porphyrin‐based sonosensitizers. The covalent modification of OPV enhanced the water solubility of 5, 10, 15, 20‐tetra (4‐aminophenyl) porphyrins and reduced the self‐aggregation of porphyrin molecules. Since the emission spectrum of OPV overlaps well with the absorption spectrum of porphyrin, the effective energy transfer from OPV (donor) to TAPP (acceptor) improves the ROS yield of the TAPP under US irradiation, which can be used as a potent agent for sonodynamic antitumor therapy.

**FIGURE 3:**
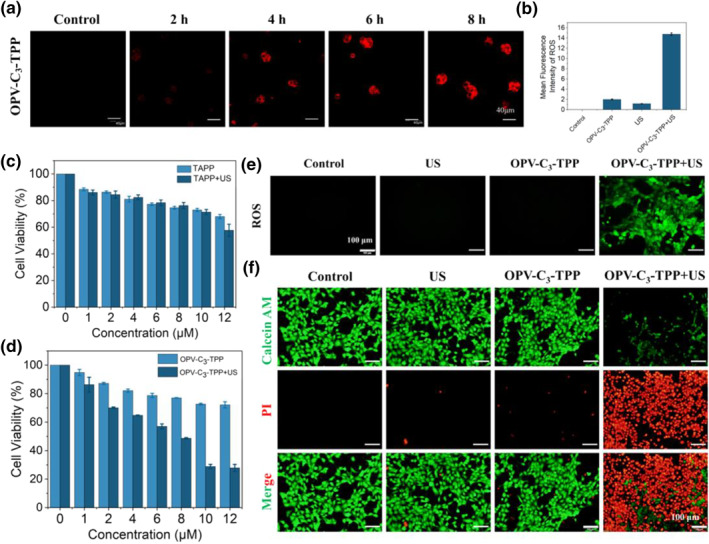
In vitro experiments of OPV‐C_3_‐TPP. (a) Fluorescence imaging of 4T1 cells after co‐incubation with OPV‐C_3_‐TPP. Scale bar = 40 μm. (b) Quantification of the fluorescence intensity of DCFH‐DA probe from (e). (c) Cell viability of 4T1 cells after co‐incubating with different concentrations of TAPP and being exposed to US for 6 min. (d) Cell viability of 4T1 cells after co‐incubating with different concentrations of OPV‐C_3_‐TPP and being exposed to US for 6 min. (e) Intracellular ROS detection using DCFH‐DA probe after being exposed to US for 5 min (DCFH: *λ*_ex_ = 488 nm, *λ*_em_ = 525–575 nm) [OPV‐C_3_‐TPP] = 10 μM. (f) Co‐staining of 4T1 cells by calcein‐AM/PI after incubation with OPV‐C_3_‐TPP for 6 h and being exposed to US for 6 min (calcein‐AM: *λ*_ex_ = 488 nm, *λ*_em_ = 500–540 nm; PI: *λ*_ex_ = 559 nm, *λ*_em_ = 570–620 nm). US conditions: 1.0 MHz, 0.5 W cm^−2^, 50% duty cycle. Scale bar = 100 μm. DCFH‐DA, 2′,7′‐dichlorodihydrofluorescein diacetate; PI, propidium iodide; ROS, reactive oxygen species; TAPP, 5, 10, 15, 20‐tetra (4‐aminophenyl) porphyrin; US, ultrasound.

By virtue of the remarkable ROS generation effect of OPV‐C_3_‐TPP under US irradiation, its SDT anti‐tumor effect at the cellular level was further investigated. The cellular uptake of OPV‐C_3_‐TPP in 4T1 cells was tested by confocal laser scanning microscopy. The red fluorescence intensity of OPV‐C_3_‐TPP in 4T1 cells became stronger with the increasing incubation time, suggesting that the cellular uptake of OPV‐C_3_‐TPP was time‐dependent. After incubation for 4 h, the uptake of probe reached the plateau (Figures 3a and S2). Furthermore, the standard methyl thiazolyl tetrazolium (MTT) assay was used to assess the biocompatibility and biosafety after 4T1 cells were incubated with OPV‐C_3_‐TPP or TAPP at different concentrations (1, 2, 4, 6, 8, 10, 12 μM) for 24 h. As shown in Figure 3c, TAPP exhibited slight cytotoxicity to 4T1 cells at a high concentration (12 μM). Compared with TAPP, OPV‐C_3_‐TPP is less cytotoxic, indicating it has better biocompatibility and biosafety for pharmaceutical applications. Notably, the irradiation of US with 6 min had no significant damage to cells (Figure S3). However, the cell viability of 4T1 consistently decreased with the increase in OPV‐C_3_‐TPP concentration under US irradiation. At the OPV‐C_3_‐TPP concentration of 12 μM, the viability of 4T1 cells was measured as little as 28% (Figure 3c,d), indicating that OPV‐C_3_‐TPP generated a high anti‐tumor cell efficiency. In contrast, under the same conditions, 60% cells still live after incubation with TAPP, suggesting that OPV‐C_3_‐TPP has a better SDT effect than TAPP. These results indicate that OPV‐C_3_‐TPP not only has better biocompatibility but also has outstanding therapeutic performance.

ROS has been recognized as a major cause of US‐mediated death of tumor cells. Accordingly, the intracellular ROS generation was assayed by using DCFH‐DA probe. The 4T1 cells were incubated with OPV‐C_3_‐TPP (10 μM) or TAPP (10 μM) for 6 h and then irradiated with US. No green fluorescence signals were detected from the control, OPV‐C_3_‐TPP without US, TAPP, TAPP + US and US‐treated cells (Figures 3b,e and S4). Comparatively, the OPV‐C_3_‐TPP + US group showed bright and strong green fluorescence due to the generation of large amounts of ROS in the cells, presenting great SDT potential (Figure 3e). These results suggested that OPV‐C_3_‐TPP is promising as a sonosensitizer for anti‐cancer.

To further validate the US‐triggered anti‐tumor ability of OPV‐C_3_‐TPP, a celcein acetoxymethyl ester/propidium iodide (calcein‐AM/PI) co‐stainning assay was performed. Here, the live cells (green fluorescence) and dead cells (red fluorescence) were visualized in the different treatment groups by fluorescence microscopy. As presented in Figure 3f, the control, US, and OPV‐C_3_‐TPP groups showed bright green fluorescence and faint red fluorescence, implying that 4T1 cells are alive in these treatment groups. In contrast, upon US treatments, the OPV‐C_3_‐TPP incubated group (OPV‐C_3_‐TPP + US group) showed bright red fluorescence of PI, illustrating the most cell death. Furthermore, as a control, green fluorescence in the TAPP + US group indicated that its cells were in good condition under the same conditions (Figure S5). These results demonstrated that OPV‐C_3_‐TPP combined with US can significantly increase the anti‐tumor cells effect, which were consistent with the aforementioned MTT data (Figure 3d).

**FIGURE 4:**
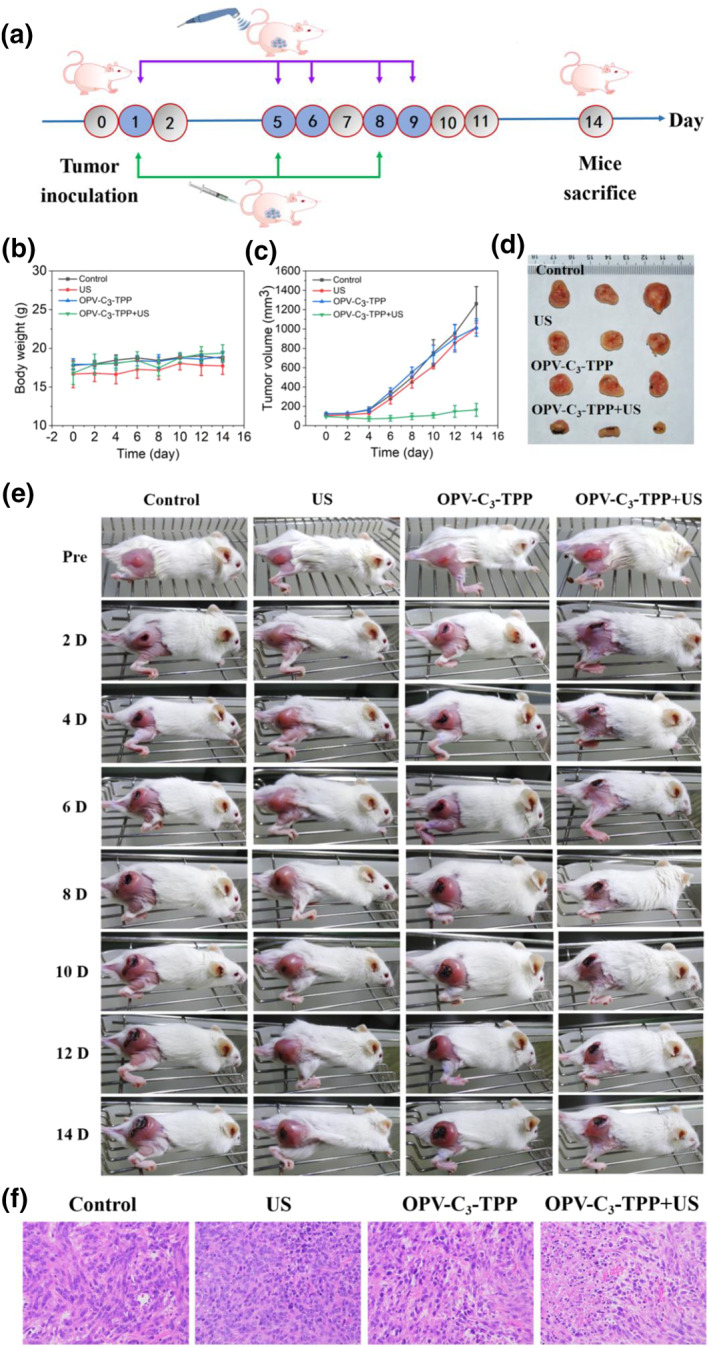
In vivo sonodynamic therapy anti‐tumor efficiency of OPV‐C_3_‐TPP. (a) Schematic illustration of in vivo treatment process of OPV‐C_3_‐TPP with US. (b) Body weight changes of mice in different treatment groups during tumor treatment. (c) Tumor volume curves after different treatments. (d) Representative photographs of tumors from different treatment groups at defined time points. (e) Photographs of the appearance of 4T1 tumor‐bearing mice from different treatment groups at defined time points. (f) H&E‐stained tumor slices from mice in different treatment groups. H&E, hematoxylin and eosin; US, ultrasound.

**FIGURE 5:**
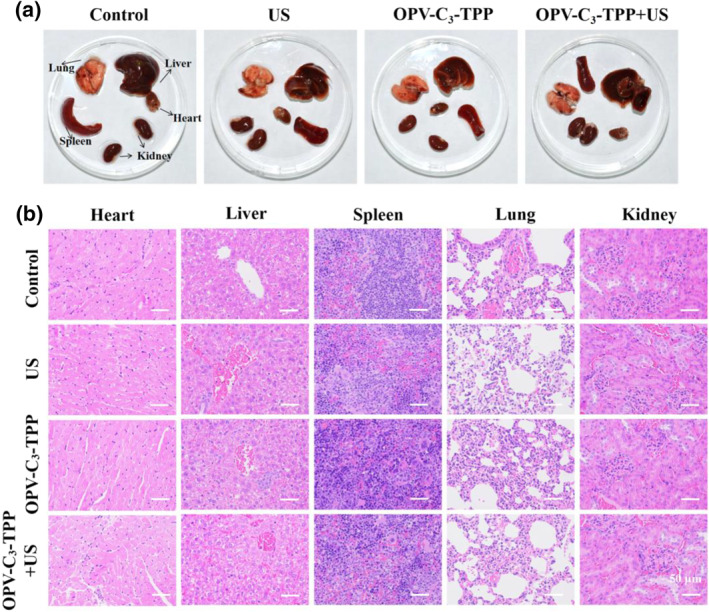
(a) Representative photographs of organs from mice in different groups after therapy for 14 days; (b) H&E‐stained organ slices collected from mice in different treatment groups. Scale bar = 50 μm. H&E, hematoxylin and eosin.

Based on the remarkable in vitro therapeutic efficacy and ideal biocompatibility of OPV‐C_3_‐TPP, we further evaluated its anti‐tumor effects in vivo. Firstly, a subcutaneous tumor model was established for evaluation by subcutaneously injecting 4T1 breast cancer cells into the upper back of the right thigh of female Balb/c mice. When the tumor volume grew up to about 100 mm^3^, 4T1 tumor‐bearing mice were randomly divided into four groups. The groups were as follows: (I) Control, (II) US only, (III) OPV‐C_3_‐TPP only, and (IV) OPV‐C_3_‐TPP + US. The US conditions were set as 1.0 MHz, 1.5 W cm^−2^, 50% duty cycle, and 5 min. These mice received three injections on days 1, 5, and 8. At post‐injection, the mice in the II and IV groups were exposed to US irradiation. On the following day, the US treatment was repeated once (Figure 4a). The body weights of the mice were measured and recorded every 2 days during the 14‐day treatment period, and there were no significant fluctuations in the body weights of the mice in each group, suggesting that these therapies had minimal effect on the health of the mice (Figure 4b). The tumor volume of the mice was also measured every 2 days, and it is noteworthy that the tumors of the mice in the control, US only and OPV‐C_3_‐TPP only groups all maintained rapid growth. However, the tumor growth was inhibited effectively in the OPV‐C_3_‐TPP + US group, confirming that the OPV‐C_3_‐TPP as a sonosensitizer could realize excellent therapeutic efficacy (Figure 4c–e). Besides, hematoxylin and eosin (H&E) stained images further validated the anti‐tumor effect. At the end of treatment, major organs and tumors from all groups were collected and stained with H&E. As shown in Figure 4f, a large number of cell necrosis and apoptosis could be observed in the OPV‐C_3_‐TPP + US group, whereas the tumor cells in the control group were morphologically intact without obvious necrosis, and the tumors of the mice in the US only and the OPV‐C_3_‐TPP only were slightly destroyed. The result was in agreement with the trends in the tumor inhibition. Furthermore, OPV‐C_3_‐TPP has good biosafety with no obvious toxic side effects on major organs (Figure 5). All the results demonstrated that OPV‐C_3_‐TPP had superior anti‐tumor effects.

## 3 CONCLUSION

In summary, we proposed and developed a novel sono‐enhanced anti‐tumor strategy to overcome the poor water solubility, easy aggregation and low ROS yield of porphyrins based on a unimolecular probe, the porphyrin derivative OPV‐C_3_‐TPP. OPV was covalently linked with TAPP to construct an excellent anti‐tumor nanoplatform. The water‐solubility was enhanced and the self‐aggregation of porphyrin was reduced by OPV groups. Furthermore, the efficient ROS production under US was obtained due to the high energy transfer from OPV to TAPP, which brought effective SDT for anti‐tumor in vitro and in vivo. Thus, this study provides a promising sonosensitizer for SDT, and puts forward a feasible strategy to improve the performance of sonosensitizers in SDT.

## CONFLICT OF INTEREST STATEMENT

The authors declare no conflicts of interest.

## ETHICS STATEMENT

All animal procedures and experimental protocols received approval from both the Shaanxi Provincial Laboratory Animal Management Committee (Xi'an, Shaanxi, China) and the Ethics Committee of Shaanxi Normal University (Xi'an, Shaanxi, China).

## Supporting information

Supporting Information S1

## Data Availability

Supporting Information is available from the Wiley Online Library or from the author.

## References

[1] R. L. Siegel , K. D. Miller , H. E. Fuchs , A. Jemal , CA Cancer J. Clin. 2021, 71, 7. 33433946 10.3322/caac.21654

[2] J. Li , R. C. Jiang , Q. Wang , X. Li , X. M. Hu , Y. Yuan , X. M. Lu , W. J. Wang , W. Huang , Q. L. Fan , Biomaterials 2019, 217, 119304. 31279099 10.1016/j.biomaterials.2019.119304

[3] J. C. Li , S. Q. Wang , F. Fontana , C. Tapeinos , M. A. Shahbazi , H. J. Han , H. A. Santos , Bioact. Mater. 2023, 23, 471. 36514388 10.1016/j.bioactmat.2022.11.013 PMC9727595

[4] H. J. Ma , R. X. Li , H. B. Meng , M. Tian , X. H. Zhang , Y. L. Liu , L. Li , J. Y. Yuan , Y. Wei , Small 2023, 19, 2204778. 10.1002/smll.202204778 36802107

[5] Z. Q. Zhang , Z. N. Lu , Q. Yuan , C. Zhang , Y. L. Tang , J. Mater. Chem. B 2021, 9, 2240. 33596297 10.1039/d0tb02996c

[6] M. Yang , H. Zhao , Z. Q. Zhang , Q. Yuan , Q. Feng , X. R. Duan , S. Wang , Y. L. Tang , Chem. Sci. 2021, 12, 11515. 34667555 10.1039/d1sc01317c PMC8447874

[7] A. G. Niculescu , A. M. Grumezescu , Appl. Sci. 2021, 11, 3626.

[8] N. Yumita , R. Nishigaki , K. Umemura , S. Umemura , Jpn. J. Cancer Res. 1989, 80, 219. 2470713 10.1111/j.1349-7006.1989.tb02295.x PMC5917717

[9] S. Liang , X. R. Deng , P. A. Ma , Z. Y. Cheng , J. Lin , Adv. Mater. 2020, 32, 2003214. 10.1002/adma.202003214 33064322

[10] X. H. Lin , J. B. Song , X. Y. Chen , H. H. Yang , Angew. Chem., Int. Ed. 2020, 59, 14212. 10.1002/anie.201906823 31267634

[11] R. Canaparo , F. Foglietta , N. Barbero , L. Serpe , Adv. Drug Delivery Rev. 2022, 189, 114495. 10.1016/j.addr.2022.114495 35985374

[12] Z. R. Gong , Z. F. Dai , Adv. Sci. 2021, 8, 2002178. 10.1002/advs.202002178 PMC8132157 34026428

[13] Y. Zhang , X. Q. Zhang , H. C. Yang , L. Yu , Y. J. Xu , A. Sharma , P. Yin , X. Y. Li , J. S. Kim , Y. Sun , Chem. Soc. Rev. 2021, 50, 11227. 34661214 10.1039/d1cs00403d

[14] Y. Pu , H. Yin , C. Dong , H. Xiang , W. Wu , B. Zhou , D. Du , Y. Chen , H. Xu , Adv. Mater. 2021, 33, e2104641. 34536041 10.1002/adma.202104641

[15] Y. Tang , E. Pang , P. Zhu , Q. Tan , S. Zhao , B. Wang , C. Yao , X. Song , M. Lan , Smart Mol. 2024, 2, e20240003. 40625906 10.1002/smo.20240003 PMC12118247

[16] Y. X. He , S. H. Liu , J. Yin , J. Yoon , Coord. Chem. Rev. 2021, 429, 213610.

[17] S. Son , J. H. Kim , X. W. Wang , C. L. Zhang , S. A. Yoon , J. Shin , A. Sharma , M. H. Lee , L. Cheng , J. S. Wu , J. S. Kim , Chem. Soc. Rev. 2020, 49, 3244. 32337527 10.1039/c9cs00648f

[18] T. Xu , S. J. Zhao , C. W. Lin , X. L. Zheng , M. H. Lan , Nano Res. 2020, 13, 2898.

[19] Z. Y. Cao , G. T. Yuan , L. L. Zeng , L. Bai , X. Liu , M. X. Wu , R. L. Sun , Z. T. Chen , Y. Jiang , Q. Y. Gao , Y. X. Chen , Y. L. Zhang , Y. Pan , J. F. Wang , ACS Nano 2022, 16, 10608. 35759554 10.1021/acsnano.2c02177

[20] Y. Harada , K. Ogawa , Y. Irie , H. Endo , L. B. Feril , T. Uemura , K. Tachibana , J. Controlled Release 2011, 149, 190. 10.1016/j.jconrel.2010.10.012 20951750

[21] X. Tan , J. T. Huang , Y. Q. Wang , S. S. He , L. Jia , Y. H. Zhu , K. Y. Pu , Y. Zhang , X. L. Yang , Angew. Chem., Int. Ed. 2021, 60, 14051. 10.1002/anie.202102703 33797161

[22] X. W. Wang , X. Y. Zhong , L. X. Bai , J. Xu , F. Gong , Z. L. Dong , Z. J. Yang , Z. J. Zeng , Z. Liu , L. Cheng , J. Am. Chem. Soc. 2020, 142, 6527. 32191455 10.1021/jacs.9b10228

[23] R. R. Zhou , M. Q. Chang , M. J. Shen , Y. Cong , Y. Chen , Y. Wang , Adv. Sci. 2023, 10, 2301764. 10.1002/advs.202301764 PMC10477905 37395421

[24] J. Ouyang , L. Deng , W. S. Chen , J. P. Sheng , Z. J. Liu , L. Q. Wang , Y. N. Liu , Chem. Commun. 2018, 54, 2874. 10.1039/c8cc00392k 29493688

[25] F. Wang , B. Y. Wang , W. You , G. Chen , Y. Z. You , Nano Res. 2022, 15, 9223. 35845146 10.1007/s12274-022-4599-5 PMC9274620

[26] T. T. Wu , Y. Liu , Y. Cao , Z. H. Liu , Adv. Mater. 2022, 34, 2110364. 10.1002/adma.202110364 35133042

[27] X. J. Xing , S. J. Zhao , T. Xu , L. Huang , Y. Zhang , M. H. Lan , C. W. Lin , X. L. Zheng , P. F. Wang , Coord. Chem. Rev. 2021, 445, 214087.

[28] M. H. Lan , S. J. Zhao , W. M. Liu , C. S. Lee , W. J. Zhang , P. F. Wang , Adv. Healthcare Mater. 2019, 8, 1900132. 10.1002/adhm.201900132 31067008

[29] A. Q. Ma , H. Q. Chen , Y. H. Cui , Z. Y. Luo , R. J. Liang , Z. H. Wu , Z. Chen , T. Yin , J. Ni , M. B. Zheng , L. T. Cai , Small 2019, 15, 1804028. 10.1002/smll.201804028 30589210

[30] Z. L. Dong , L. Z. Feng , Y. Hao , Q. G. Li , M. C. Chen , Z. J. Yang , H. Zhao , Z. Liu , Chem 2020, 6, 1391.

[31] T. Chen , W. W. Zeng , Y. Q. Liu , M. Yu , C. Y. Huang , Z. Q. Shi , C. C. Lin , J. Tang , L. Mei , M. Y. Wu , Small 2022, 18, 2202964. 10.1002/smll.202202964 35717674

[32] T. Y. Tong , H. Q. Lei , S. Q. Zhang , D. G. Jiang , Y. P. Guan , C. Y. Xing , H. K. Chen , X. W. Yang , Y. Kang , J. Pang , Adv. Healthcare Mater. 2022, 11, 2201472. 10.1002/adhm.202201472 36126678

[33] Y. T. Zhao , Z. Q. Zhang , Z. N. Lu , H. Wang , Y. L. Tang , ACS Appl. Mater. Interfaces 2019, 11, 38467. 31553165 10.1021/acsami.9b12375

[34] Y. Chen , Q. Tan , Y. Tang , E. Pang , R. Peng , M. Lan , D. Bai , Biomater. Sci. 2024, 12, 1864. 38411494 10.1039/d3bm01994b

[35] P. Huang , X. Q. Qian , Y. Chen , L. D. Yu , H. Lin , L. Y. Wang , Y. F. Zhu , J. L. Shi , J. Am. Chem. Soc. 2017, 139, 1275. 28024395 10.1021/jacs.6b11846

[36] H. Q. Chen , L. L. Liu , A. Q. Ma , T. Yin , Z. Chen , R. J. Liang , Y. Z. Qiu , M. B. Zheng , L. T. Cai , Biomaterials 2021, 269, 120639. 33434714 10.1016/j.biomaterials.2020.120639

[37] H. Xu , N. Yu , J. L. Zhang , Z. J. Wang , P. Geng , M. Wen , M. Q. Li , H. J. Zhang , Z. G. Chen , Biomaterials 2020, 257, 120239. 32736261 10.1016/j.biomaterials.2020.120239

[38] M. Wen , N. Yu , S. W. Wu , M. M. Huang , P. Qiu , Q. Ren , M. F. Zhu , Z. G. Chen , Bioact. Mater. 2022, 18, 242. 35387175 10.1016/j.bioactmat.2022.03.009 PMC8961299

[39] Q. H. Feng , W. X. Zhang , X. M. Yang , Y. Z. Li , Y. W. Hao , H. L. Zhang , L. Hou , Z. Z. Zhang , Adv. Healthcare Mater. 2018, 7, 1700957.

[40] P. H. Zhao , Y. L. Wu , X. Y. Li , L. L. Feng , L. Zhang , B. Y. Zheng , M. R. Ke , J. D. Huang , Angew. Chem., Int. Ed. 2022, 61, e202113506. 10.1002/anie.202113506 34761489

[41] C. T. Deng , M. C. Zheng , S. P. Han , Y. J. Wang , J. Q. Xin , O. Aras , L. Cheng , F. F. An , Adv. Funct. Mater. 2023, 33, 2300348. 38045635 10.1002/adfm.202300348 PMC10691834

[42] X. Qu , F. Yin , M. M. Pei , Q. Chen , Y. Y. Zhang , S. W. Lu , X. L. Zhang , Z. Y. Liu , X. Y. Li , H. R. Chen , Y. Zhang , H. L. Qin , ACS Nano 2023, 17, 11466. 37201179 10.1021/acsnano.3c01308 PMC10311605

[43] X. R. Song , Q. Zhang , M. Q. Chang , L. Ding , H. Huang , W. Feng , T. M. Xu , Y. Chen , Adv. Mater. 2023, 35, 2212259. 10.1002/adma.202212259 36812400

[44] F. F. Yang , J. Dong , Z. F. Li , Z. H. Wang , ACS Nano 2023, 17, 4102. 36802411 10.1021/acsnano.2c10251

[45] H. R. Lin , H. T. Bai , Z. W. Yang , Q. Shen , M. Y. Li , Y. M. Huang , F. T. Lv , S. Wang , Chem. Commun. 2022, 58, 7232. 10.1039/d2cc02177c 35707996

[46] H. Sun , K. S. Schanze , ACS Appl. Mater. Interfaces 2022, 14, 20506. 35473368 10.1021/acsami.2c02475

[47] Y. Y. Wu , C. Y. Shi , G. B. Wang , H. Sun , S. Y. Yin , J. Mater. Chem. B 2022, 10, 2995. 35393982 10.1039/d1tb02816b

[48] Z. L. Zeng , C. Zhang , S. S. He , J. T. Li , K. Y. Pu , Adv. Mater. 2022, 34, 2203246. 10.1002/adma.202203246 35524454

[49] X. Wang , M. Wu , H. Z. Li , J. L. Jiang , S. S. Zhou , W. Z. Chen , C. Xie , X. Zhen , X. Q. Jiang , Adv. Sci. 2022, 9, 2104125. 10.1002/advs.202104125 PMC8867194 34989170

