# Supplementary material for: Conjugated oligo (phenylene vinylene) covalently linked porphyrin for sonodynamic therapy
Source: Smart Mol. 2024 Nov 13;3(2):e20240035. doi: 10.1002/smo.20240035 (PMC12262008; doi:10.1002/smo.20240035)
Supplement: Supplementary file 1 — Supporting Information S1 [file SMO2-3-e20240035-s001.docx]

Conjugated oligo (phenylene vinylene) covalently linked porphyrin for sonodynamic therapy

Wenhua Jia, Junqing Wang, Ling Li, Qiong Yuan, Yuze Wang, Xinyi Zhang, Yanli Tang*

Key Laboratory of Analytical Chemistry for Life Science of Shaanxi Province, Key Laboratory of Applied Surface and Colloid Chemistry, Ministry of Education, School of Chemistry and Chemical Engineering, Shaanxi Normal University, Xi'an 710119, P. R. China

E-mail: [yltang@snnu.edu.cn](mailto:yltang@snnu.edu.cn)

**Contents**

[**Experimental Section S-3**](#_Toc11505)

[**Materials and characterization. S-3**](#_Toc18426)

[**In vitro ROS generation S-3**](#_Toc30768)

[**Cellular experiments S-4**](#_Toc22481)

[**Cellular uptake S-4**](#_Toc19350)

[**Cell cytotoxicity S-4**](#_Toc19961)

[**Intracellular ROS generation S-5**](#_Toc11432)

[**Live/Dead Cell Staining Assay S-5**](#_Toc8773)

[**4T1 tumor xenograft establishment S-6**](#_Toc734)

[**In vivo antitumor efficacy S-6**](#_Toc31093)

[**Supporting Figures S-7**](#_Toc30330)

# Experimental Section

## Materials and characterization.

All chemical reagents were commercially obtained from J&K Chemical Ltd. and Aladdin Industrial Corporation and directly used without further purification except special instruction. The fluorescent probes used in cell imaging experiments Calcein AM, PI were purchased from Proteintech Biotechnology Co., Ltd. Other experimental reagents are purchased from Shanghai Biotech, Sigma-Aldrich, Gibco, and can be used directly without any special instructions. The NMR spectra were measured on Bruker Ascend 300, 400 or 600 MHz spectrometers. UV−vis absorption spectra were taken by SHIMADZU UV-2600 spectrophotometer. Fluorescence spectra were taken by Hitachi F-7000 spectrophotometer. The absorbance for MTT analysis was measured by a microplate reader (Spectramax M5). Confocal laser scanning microscopy images of 4T1 cells were obtained on Olympus Fluoview 1200. The images were acquired by Olympus IX73 fluorescence microscopy. All ultrasound experiments were studied with the DJO-2776.

## In vitro ROS generation

For ROS detection, ROS generation was measured by 2’,7’-dichlorodihydro-fluorescein diacetate (DCFH-DA), which is converted to non-fluorescent DCFH under alkaline conditions. 2 μL of OPV-C_3_-TPP (1 mM) solution was added to 1998 μL DCFH (40 μM). Then, the mixture was exposed to US irradiation (1.0 MHz, 0.5 W cm^-2^, 50% duty cycle) for different durations. The fluorescence spectrophotometer was used to measure the fluorescence intensity of the mixture (λ_ex_: 488 nm, λ_em_: 525 nm). DCFH solution without OPV-C_3_-TPP as control group. All experiments were conducted three times in parallel.

## Cellular experiments

Mouse breast cancer cells (4T1) were cultured in RPMI 1640 (Gibco), 10% fetal bovine serum (FBS, Four Seasons), 1% penicillin-streptomycin (Gibco) cell culture medium at 37 ^o^C under 5% CO_2_.

## Cellular uptake

4T1 cells seeded in confocal dish (3×10^5^ cells per dish) and incubated for 12 h. After the cells were observed to be fully adherent to the wall, the original medium was discarded and the cell were washed three times with PBS buffer solution (10 mM, pH 7.4). Then, OPV-C_3_-TPP (5 μM, 1 mL) was added and incubated for different time periods (2, 4, 6, 8 h). At the end of incubation, the cells were washed three times with PBS, Finally, the cells were visualized with Confocal Laser Scanning Microscope (CLSM) for monitoring the cellular uptake of OPV-C_3_-TPP.

## Cell cytotoxicity

4T1 cells were seeded in 96-wells plates (9×10^3^ cells per well) and incubated for 24 h, The medium of the 96-well plate was discarded. Subsequently, complete medium containing TAPP or OPV-C_3_-TPP at a serial concentration of 1, 2, 4, 6, 8, 10, 12 μM were added to 96-well plate, followed by incubation for 24 h. For sonocytotoxicity, 4T1 cells were treated with US irradiation (1.0 MHz, 0.5 W cm^-2^, 50% duty cycle) for 6 min after co-incubated with OPV-C_3_-TPP for 4 h and continued to be incubated for 20 h. Then, Methyl thiazolyl tetrazolium (5 mg/mL, 10 μL) was added to each well, and the incubation was continued for 4 h, the medium was discarded. Methyl thiazolyl tetrazolium (MTT) assay was performed according to a standard protocol to determine cell viabilities.

## Intracellular ROS generation

4T1 cells were seeded in 24-wells plates (6×10^4^ cells per well) and incubated for 24 h. The experiments were randomly divided into the following groups: i) control group (without any treatment); ii) US group (1.0 MHz, 0.5 W cm^-2^, 50% duty cycle, 5 min); iii) OPV-C_3_-TPP group (10 μM); and iv) OPV-C_3_-TPP+US group (10 μM). Then, the cells were treated with RPMI 1640 containing OPV-C_3_-TPP (10 μM), After incubation for 6 h, the cells in US groups were exposed to US irradiation (1.0 MHz, 0.5 W cm^-2^, 50% duty cycle) for 5 min, the media were replaced by RPMI 1640 containing 2',7'-dichlorodihydrofluorescein diacetate (DCFH-DA, 20 μM) and incubated for 30 min. Then, the cells were washed with PBS three times, and the images were acquired by fluorescence microscopy. In addition, as a control, the production of ROS in cells of TAPP under the same conditions was detected.

## Live/Dead Cell Staining Assay

4T1 cells were seeded in 24-wells plates (1×10^5^ cells per well) and incubated for 12 h. After different treatments, Control; US only: 1.0 MHz, 0.5 W cm^-2^, 50% duty cycle, 6 min; OPV-C_3_-TPP only: 10 μM; OPV-C_3_-TPP + US: 10 μM, 1.0 MHz, 0.5 W cm^-2^, 50% duty cycle, 6 min. 4T1 cells were incubated with Calcein-AM (4 μM) and propidium (9 μM) for 30 min. After being washed by PBS, the images were acquired by fluorescence microscopy. In addition, as a control, we examined apoptosis and necrosis caused by TAPP under the same conditions.

## 4T1 tumor xenograft establishment

All animal procedures and experimental protocols received approval from both the Shaanxi Provincial Laboratory Animal Management Committee (Xi’an, Shaanxi, China) and the Ethics Committee of Shaanxi Normal University (Xi’an, Shaanxi, China). SPF BALB/c female mice (5 weeks) were purchased from Shaanxi Normal University Animal Experiment Center. All the experiments on mice were performed following local ethics committee. 1×10^6^ 4T1 cells were subcutaneously injected into the upper back of the right thigh of female mice for establishing the xenograft tumor model. When the tumor became distinct and the tumor volume reached ~100 mm^3^, these mice were used for subsequent experiments.

## In vivo antitumor efficacy

4T1 tumor-bearing mice was divided into four groups (n = 3) at random: i) control group with only PBS injection; ii) US group (1.0 MHz, 1.5 W cm^-2^, 50% duty cycle, 5 min); iii) OPV-C_3_-TPP group (100 μM); iv) OPV-C_3_-TPP + US group (100 μM, 1.0 MHz, 1.5 W cm^-2^, 50% duty cycle, 5 min). These mice received three injections on day 1, 5, and 8. At post-injection, the mice in US-containing groups were exposed to US irradiation (1.0 MHz, 1.5 W cm^-2^, 50% duty cycle, 5 min), which was repeated once on the following day. The length and width of tumors and body weights of mice were measured and recorded every 2 days. The tumor volume was determined by the following equation: volume = width^2^×length /2. 14 days later, the mice were sacrificed, tumors together with major organs (heart, liver, spleen, lung, and kidney) collected from all mice were sliced and fixed for H&E staining.

# Supporting Figures:


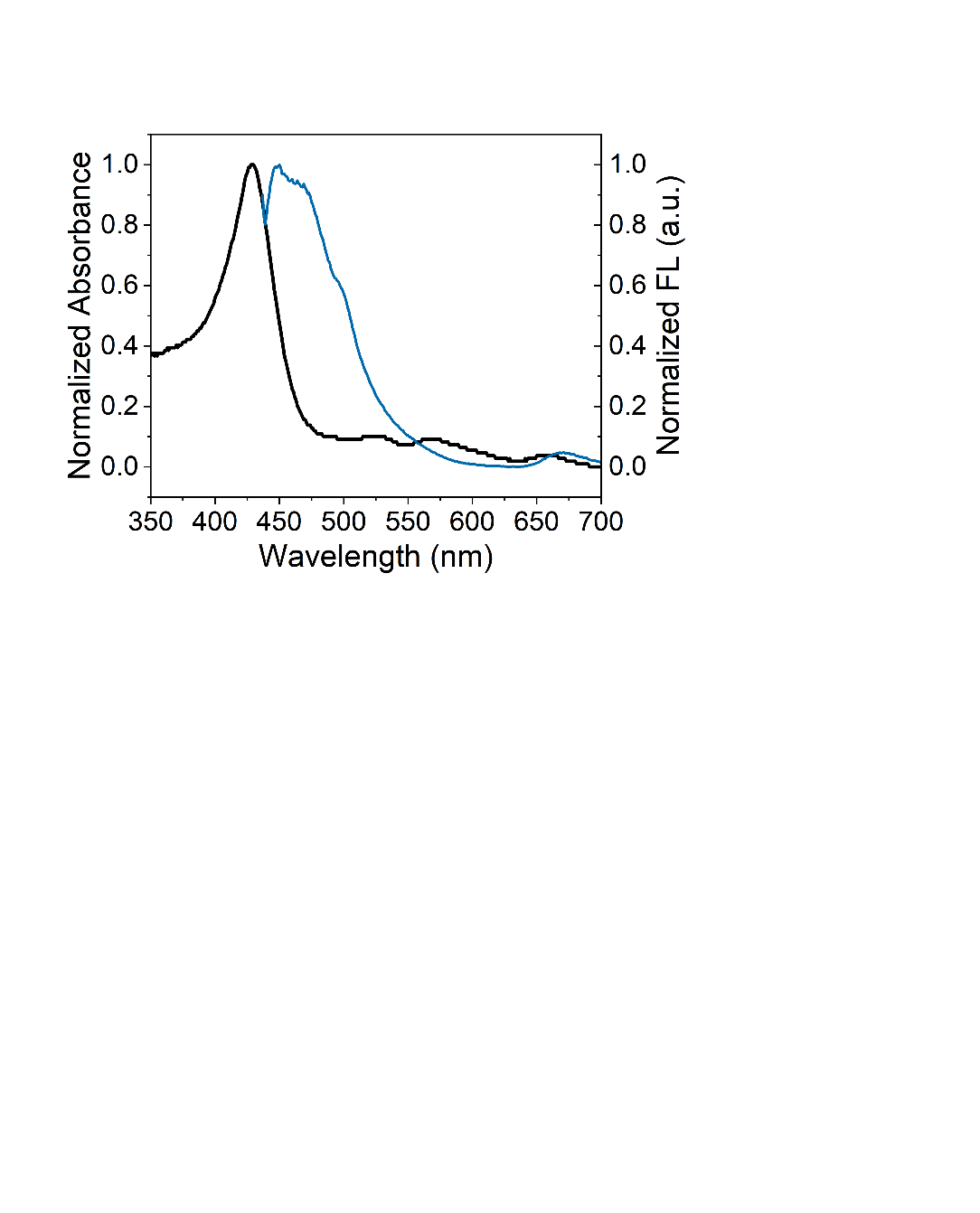


**Figure S1.** The normalized absorption and fluorescence spectra of OPV-C_3_-TPP in water. The concentration of the OPV-C_3_-TPP used to measure the absorption is 10 μM, the concentration used to measure the emission is 1 μM, and the excitation wavelength is 427 nm.


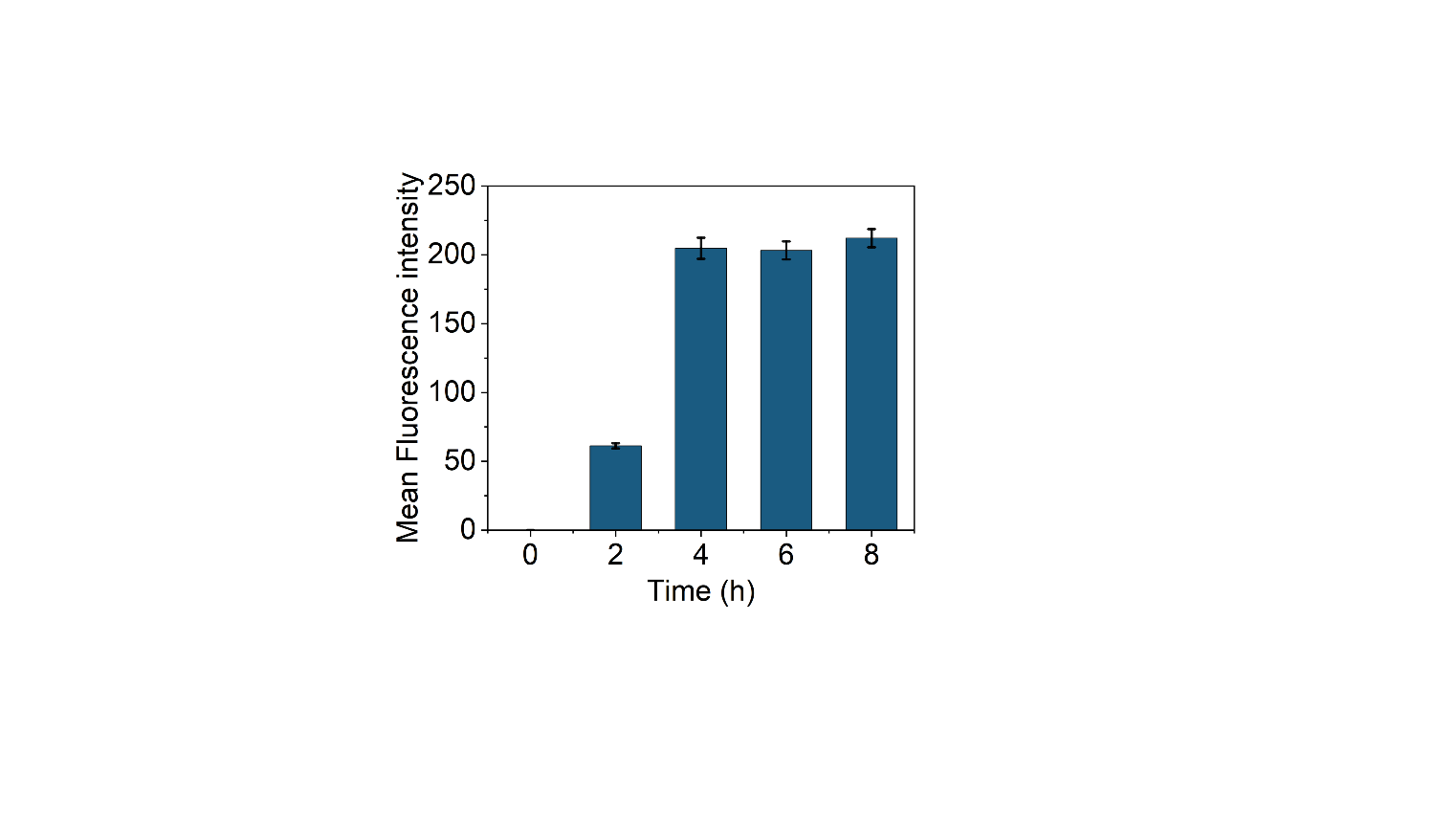


**Figure S2.** Quantification of the fluorescence intensity of OPV-C_3_-TPP inside 4T1 cells for different time.


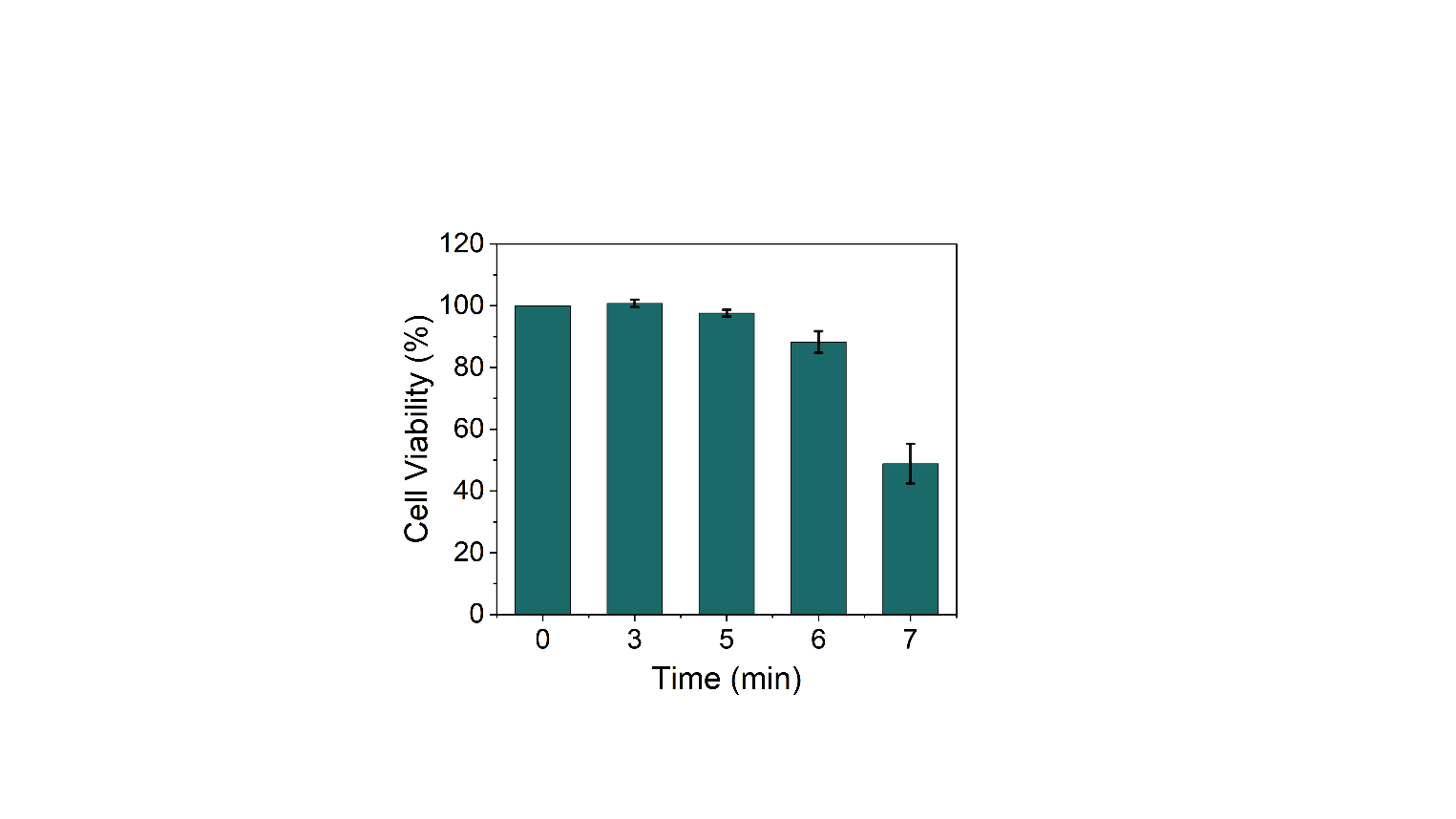


**Figure S3.** Cell viabilities of 4T1 cells after exposure to US (1.0 MHz, 0.5 W cm^-2^, 50% duty cycle) at different time point.


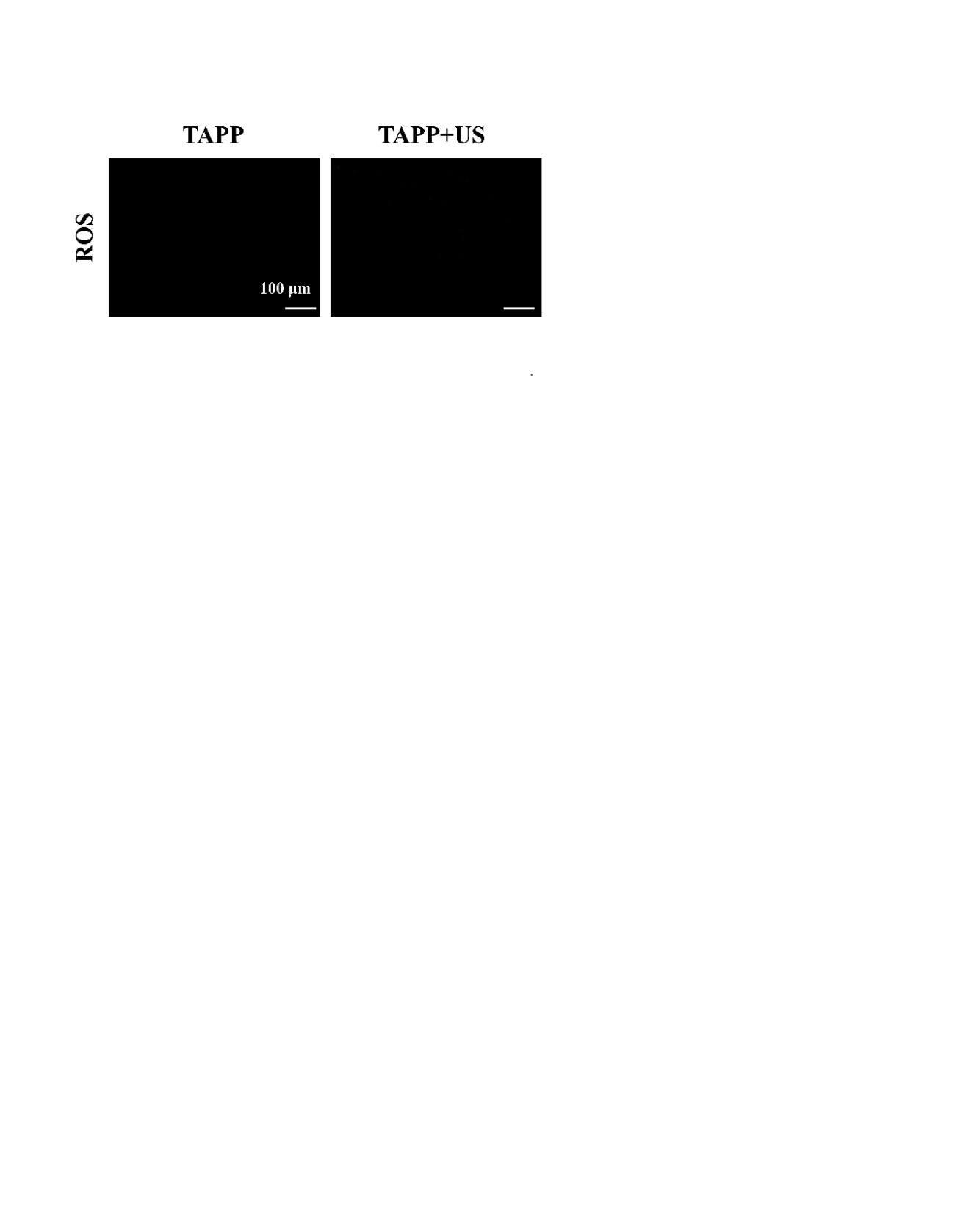


**Figure S4.** Intracellular ROS detection. US conditions: 1.0 MHz, 0.5 W cm^-2^, 50% duty cycle, 5 min (DCFH: λ_ex_ = 488 nm, λ_em_ = 525-575 nm). [TAPP] = 10 μM.


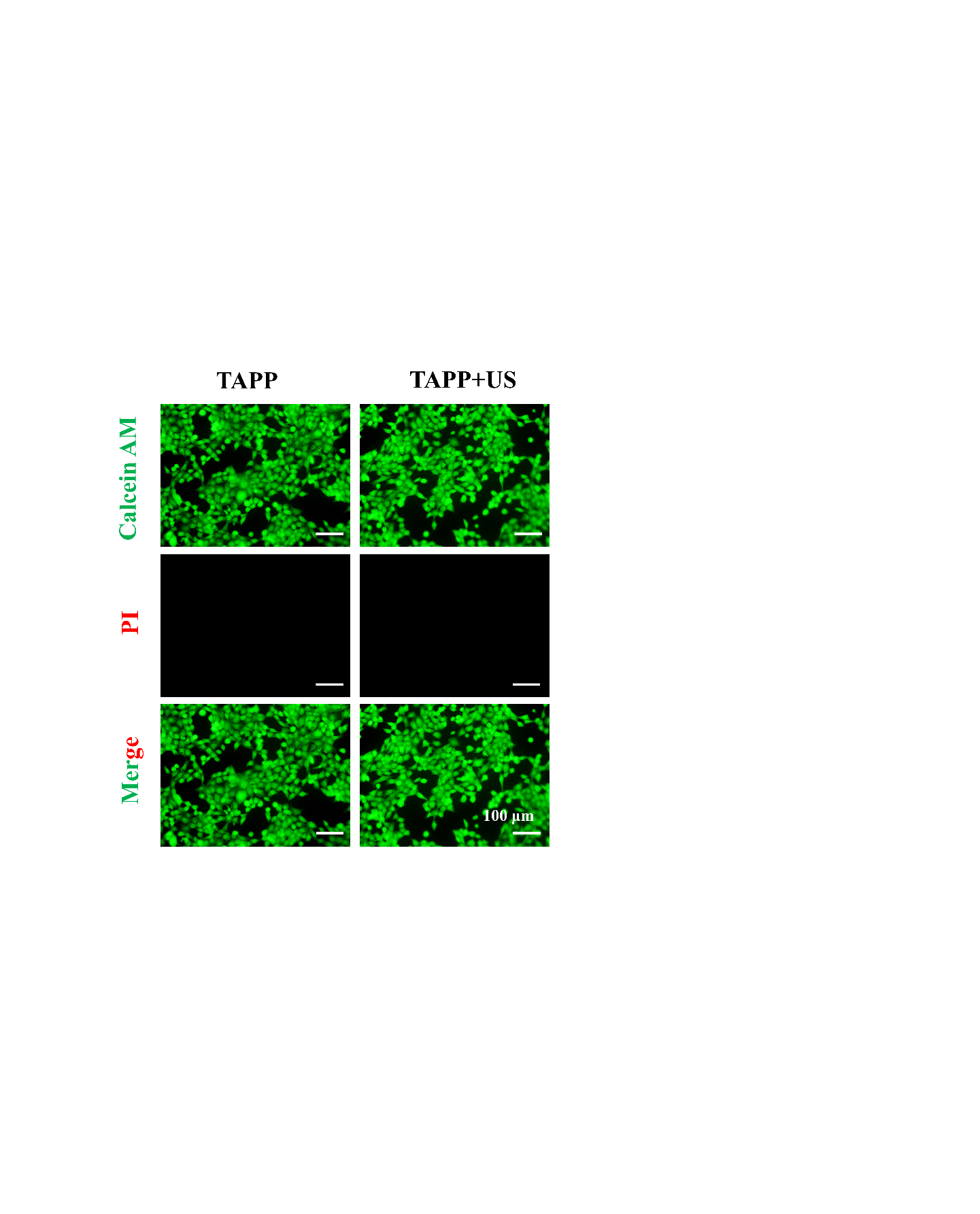


**Figure S5.** Co-staining of 4T1 cells by calcein-AM/PI incubated with TAPP for 6 h at 10 μM under US irradiation. US conditions: 1.0 MHz, 0.5 W cm^-2^, 50% duty cycle, 6 min. Scale bar = 100 μm. The fluorescence images of calcein AM and PI were collected at 500-540 nm (λex: 488 nm) and 570-620 nm (λex: 559 nm), respectively.
